# Non-Invasive Prenatal Testing of Trisomy 18 by an Epigenetic Marker in First Trimester Maternal Plasma

**DOI:** 10.1371/journal.pone.0078136

**Published:** 2013-11-01

**Authors:** Da Eun Lee, Shin Young Kim, Ji Hyae Lim, So Yeon Park, Hyun Mee Ryu

**Affiliations:** 1 Laboratory of Medical Genetics, Medical Research Institute, Cheil General Hospital and Women’s Healthcare Center, Seoul, Korea; 2 Department of Obstetrics and Gynecology, Cheil General Hospital and Women’s Healthcare Center, Kwandong University College of Medicine, Seoul, Korea; University of Bristol, United Kingdom

## Abstract

**Background:**

Quantification of cell-free fetal DNA by methylation-based DNA discrimination has been used in non-invasive prenatal testing of fetal chromosomal aneuploidy. The *maspin* (Serpin peptidase inhibitor, clade B (ovalbumin), member 5; *SERPINB5*) gene, located on chromosome 18q21.33, is hypomethylated in the placenta and completely methylated in maternal blood cells. The objective of this study was to evaluate the accuracy of non-invasive detection of fetal trisomy 18 using the unmethylated-*maspin* (U-*maspin*) gene as a cell-free fetal DNA marker and the methylated-*maspin* (M-*maspin*) gene as a cell-free total DNA marker in the first trimester of pregnancy.

**Methodology/Principal Findings:**

A nested case-control study was conducted using maternal plasma collected from 66 pregnant women, 11 carrying fetuses with trisomy 18 and 55 carrying normal fetuses. Median U-*maspin* concentrations were significantly elevated in women with trisomy 18 fetuses compared with controls (27.2 vs. 6.7 copies/mL; *P<*0.001). Median M-*maspin* concentrations were also significantly higher in women with trisomy 18 fetuses than in controls (96.9 vs. 19.5 copies/mL, *P<*0.001). The specificities of U-*maspin* and M-*maspin* concentrations for non-invasive fetal trisomy 18 detection were 96.4% and 74.5%, respectively, with a sensitivity of 90.9%.

**Conclusions:**

Our results suggest that U-*maspin* and M-*maspin* concentrations may be useful as potential biomarkers for non-invasive detection of fetal trisomy 18 in the first trimester of pregnancy, irrespective of the sex and genetic variations of the fetus.

## Introduction

Trisomy 18, also known as Edward syndrome, is the second most common form of chromosomal aneuploidy with an average incidence of 1 in 6,000 pregnancies and is caused by the presence of an extra copy of all or part of chromosome 18. Clinical features include mental retardation, failure to thrive, cardiac and kidney problems, and other congenital abnormalities. The prevalence of trisomy 18 increases in a maternal age-dependent manner, similar to the relationship already reported for Down syndrome [1]. Non-invasive prenatal screening of trisomy 18 is currently based on the use of ultrasound scans combined with maternal serum biochemical markers in the first and second trimesters. Although prenatal screening tests have greatly improved in the past decade, the best screening tests for trisomy 18 have a detection rate of 90.9% and false-positive rates of 2% in the first trimester [2]. Moreover, normal pregnant women with false-positive results undergo unnecessary invasive prenatal diagnostic procedures such as amniocentesis and chorionic villus sampling, which carry a risk of procedure-associated fetal loss [3]. Therefore, there is an urgent need for the development of accurate, stand-alone non-invasive strategies.

Cell-free fetal DNA and cell-free total DNA in maternal blood have been proposed as potential markers for non-invasive prenatal testing (NIPT) and monitoring of maternal and fetal conditions. In previous studies, cell-free fetal DNA and cell-free total DNA increased in association with various maternal and fetal complications including pre-eclampsia, preterm labor, intrauterine fetal death, fetal RhD status, single gene disorders, and fetal chromosomal aneuploidies [4]–[10].

Recently, epigenetic differences between placental and maternal cells have been explored as a promising strategy for the NIPT of fetal aneuploidies by analysis of fetal nucleic acid in maternal plasma. Because the cell-free fetal DNA in the maternal plasma is derived from the placental trophoblast cells and the cell-free maternal DNA in the maternal plasma is derived from the maternal hematopoietic cells, this epigenetic strategy has led to the identification of DNA sequences which are methylated differently in placenta and maternal blood [11], [12]. Up to now, a large number of candidate fetal epigenetic markers have been developed [13]. However, the number of markers that have been validated for detection and fetal-specificity in maternal plasma is relatively limited. The placental-derived *maspin* (Serpin peptidase inhibitor, clade B (ovalbumin), member 5; *SERPINB5*) gene was first identified as a fetal-specific marker in maternal plasma that allowed the measurement of cell-free fetal DNA concentrations in pregnancy-associated disorders, regardless of fetal sex and genetic variations [14]. The *maspin* gene is located on chromosome 18q21.33 and is a tumor suppressor gene that is differentially expressed during human placental development [15]. Chim *et al*. found that *maspin* gene is hypomethylated in the placenta and completely methylated in maternal blood [14]. Subsequently, Tong *et al*. reported that by quantification of *maspin* located on chromosome 18, the overrepresentation of chromosome 18 in maternal plasma contributed by a trisomy 18 fetus can be detected [16].

The objective of this study was to evaluate the accuracy of non-invasive detection of fetal trisomy 18 using the unmethylated-*maspin* (U-*maspin*) gene as a cell-free fetal DNA marker and the methylated-*maspin* (M-*maspin*) gene as a cell-free total DNA marker in maternal plasma during the first trimester of pregnancy.

## Materials and Methods

### Study Participants and Sample Collection

This study was conducted according to the principles expressed in the Declaration of Helsinki. Ethical approval was obtained from the Institutional Review Board and the Ethics Committee of Cheil General Hospital (CGH-IRB-2008-07). Written informed consent was obtained from each participant before blood draws for the collection of samples. We performed a nested case-control study of women who enrolled in the Cheil General Hospital Non-invasive Prenatal Testing (CNPT) program. Participants were women who received prenatal care at Cheil General Hospital and were recruited into the CNPT from October 2008 to November 2012 for NIPT of rare and incurable fetal diseases. Placental samples were collected during the first trimester by chorionic villus sampling during conventional prenatal diagnostic procedures and maternal peripheral blood samples were collected from all women. For the current study, 66 women were selected from a larger sample of 793 women enrolled in the CNPT according to criteria described below. All pregnancies were singletons at or before 14 weeks of gestation.

The case group consisted of 11 women who were subsequently detected to be carrying a trisomy 18 fetus by chorionic villus sampling for fetal karyotyping. The control group contained 55 women who delivered healthy normal neonates at term (37 weeks of gestation or more) without medical or obstetric complications, such as hypertension, diabetes, renal insufficiency, congenital anomalies, or fetal demise. Before maternal blood sampling, ultrasonography was recommended to establish the viability of each singleton pregnancy and to confirm gestational age calculated from the time of last menstruation. Maternal, fetal, and infant records were collected prospectively and maintained in an electronic database. None of the participants had histories of pre-existing hypertension, diabetes mellitus, liver disease, or chronic kidney disease.

### Sample Processing and DNA Extraction

Maternal peripheral blood samples (10 mL) were collected in EDTA tubes and immediately centrifuged at 1,600 *g* for 10 min at 4°C. The supernatant plasma was re-centrifuged at 16,000 *g* for 10 min at 4°C and aliquotted into 1-mL samples for DNA extraction. The peripheral blood cell portion was re-centrifuged at 2,500 *g* for 10 min to remove any residual plasma. All samples were stored at −80°C until analysis. Genomic DNA was extracted from placental tissues and peripheral blood cells with the QIAamp DNA Mini kit (Qiagen, Hilden, Germany). Circulating fetal DNA was extracted from 1 mL of maternal plasma using the QIAamp DSP Virus Kit (Qiagen).

### Bisulfite Sequencing of *Maspin* Promoter

Extracted genomic DNA was bisulfite-converted using the EpiTect Bisulfite kit (Qiagen) according to the manufacturer’s instructions. Bisulfite converts unmethylated cytosine into uracil while leaving methylated cytosine unchanged [17]. The methylation status of the *maspin* gene promoter in the placental tissues and maternal blood cells was determined by bisulfite sequencing. Primers were designed according to the CpG islands of the sense strand of the *maspin* gene (GenBank accession no. NT_025028.14) and are listed in Table 1.

**Table 1:** Oligonucleotide sequences.

Assay		Sequence (5′ to 3′)
Bisulfite sequencing	Forward primer	GAATGGAGATTAGAGTATTTTTTGTGTTAT
	Reverse primer	ACTTCCAAAAAACCTCCAACATAT
U-*maspin* qMSP	Forward primer	TGGTTTTGTGTGGGTTGAGAG
	Reverse primer	GGCCGGACTATAAATTACATACATACA
	Hydrolysis probe	VIC-ATTGTTGTATGTATGTTTG-MGBNFQ
	Amplicon (64bp)	TGGTTTTGTGTGGGTTGAGAGGATTGTTGTATGTATGTTTGTATGTATGTA TGTAATTTATAGT
M-*maspin* qMSP	Forward primer	CGGTTTTGCGTGGGTCGAGAG
	Reverse primer	GGCCGGACTATAAATTACATACATACG
	Hydrolysis probe	VIC-ATTGTCGTACGTATGTTT-MGBNFQ
	Amplicon (64bp)	CGGTTTTGCGTGGGTCGAGAGGATTGTCGTACGTATGTTTGTACGTATGTA TGTAATTTATAGT

PCR was performed in a total volume of 50 µL containing 1×buffer, 0.25 mM dNTPs, 1.5 mM MgCl_2_, 0.25 U Taq polymerase, 10 pM primers, and 1 µL genomic DNA. The thermal profile consisted of an initial denaturation step of 94°C for 5 min followed by 35 cycles of 94°C for 45 sec, 60°C for 45 sec, and 72°C for 45 sec, with a final extension at 60°C for 30 min.

After PCR amplification, PCR products were purified using a PCR purification kit (Bioneer, Daejeon, Korea) and sequenced using a PRISM BigDye Terminator Cycle Sequencing Kit (Applied Biosystems, Foster City, CA, USA). Sequencing products were analyzed using an ABI 3130×l Genetic Analyzer (Applied Biosystems) and electropherogram traces were interpreted with DNA sequencing analysis software version 5.3 (Applied Biosystems).

### Real-Time Quantitative Methylation-Specific PCR (qMSP)

Concentrations of U-*maspin* and M-*maspin* promoter DNA sequences (GenBank accession no. NT_025028.14, chr18∶61143918-61143981; UCSC Genome Browser Assembly GRCh37/hg19) were measured by qMSP assays using a CFX96 Real Time System (Bio-Rad, Hercules, CA, USA).

DNA extracted from 1 mL of maternal plasma was bisulfite-converted using the EZ DNA Methylation Kit (Zymo Research, Orange, CA, USA) according to the manufacturer’s protocol. The modified DNA was amplified using two different sets of primers for U-*maspin* and M-*maspin* (Table 1). The 5′ ends of the reverse primers were modified by the addition of GGCCGG to enhance specificity and sensitivity of the qMSP assays.

PCR was performed in a total volume of 25 µL containing 12.5 mL iQ Supermix, 200 nM primers, 400 nM hydrolysis probe, and 5 µL converted DNA. The thermal profile consisted of an initial denaturation step of 95°C for 10 min followed by 50 cycles of 95°C for 15 sec, 58°C for 30 sec, and 72°C for 30 sec. Calibration curves were prepared for each assay using serial dilutions of single-stranded synthetic DNA oligonucleotides specific to the U-*maspin* and M-*maspin* amplicons (Applied Biosystems). Each standard was amplified in triplicate and was included on every PCR plate. In the standard curves, slope values and r^2^ values were −3.543 and 0.994 for U-*maspin* and −3.338 and 0.997 for M-*maspin*, respectively. The PCR efficiency was calculated from the slope of the curve using the following formula: Efficiency = 10−(1/slope)−1. Both U-*maspin* and M-*maspin* assays amplified with almost optimal efficiencies of 98%.

The factor concentrations are expressed as copies/mL, and the standard factor of 6.6 pg was used to convert the data to copy number, as described previously [10]. All samples were amplified in triplicate and the final data reflect the average of the results. The average intra-assay coefficients of variation were 2.1% for U-*maspin* and 2.4% for M-*maspin*. Strict precautions were taken against contamination, and multiple negative-control water blanks were included in every analysis.

### Limit of Detection for qMSP

The mean fetal DNA concentration in maternal plasma during early pregnancy was previously estimated to be 25.4 copies/ml (range 3.3–69.4) [18]. In the qMSP assay of the *maspin* gene, 1 ml of maternal plasma was extracted, eluted in a final volume of 30 µL, and 5 µL of this sample was used for each PCR. Thus, each PCR sample contained more than four copies of the fetal DNA. The PCR is sensitive enough because more than three copies of the target are required for real-time PCR according to the minimum information for publication of quantitative real-time PCR experiments (MIQE) guidelines [19].

### Statistical Analysis

The clinical characteristics of the study population were compared between cases and controls using Student’s *t* test for continuous variables and Fisher’s exact test for categorical variables. A comparison of U-*maspin* and M-*maspin* concentrations between the two groups was performed using the Mann-Whitney *U* test. The accuracy for detecting fetal trisomy 18 using U-*maspin* and M-*maspin* concentrations was determined using the fetal karyotyping results. Receiver operating characteristic (ROC) curve analysis was performed to assess the optimal cutoff value of the factors for detecting fetal trisomy 18. The sensitivity, specificity, positive predictive value (PPV), negative predictive value (NPV), and odds ratio (OR) were calculated with 95% confidence intervals (CI) using the EpiMax Table Calculator (http://www.healthstrategy.com/epiperl/epiperl.htm). Overall accuracy was estimated according to the area under the ROC curve (AUC). Multiple logistic regression analysis was used to estimate the values of U-*maspin* or M-*maspin* concentration that were risk factors for fetal trisomy 18, controlling for potential confounding factors including maternal age, body mass index, and gestational age at the time of blood sampling. Adjusted OR and the 95% CI were calculated. A *P* value <0.05 was considered statistically significant. Statistical analysis was performed using the Statistical Package for Social Sciences version 12.0 (SPSS, Chicago, IL, USA).

## Results

The clinical and demographic characteristics of the study groups are presented in Table 2. The maternal age was higher in the case group than in the control group (median age 37 vs. 32 years, *P*<0.01). The gestational age at the time of maternal blood sampling was matched in case and control groups (median 12.4 vs. 12.3 weeks). There were no statistical differences in gravidity, nulliparity, body mass index (BMI), fetal sex ratio, and gestational age between the study groups (*P*>0.05 for each comparison). The percentage of pregnant women aged 35 years or older was higher in the case group (73%) than in the control group (18%). Interestingly, the percentage of pregnant women who underwent IVF treatment was higher in the case group (46%).

**Table 2:** Clinical characteristics in cases and controls.

Characteristics	Case (n = 11)	Control (n = 55)	*P*
At blood sampling			
Maternal age (years)			
<35	3 (27.3)	44 (80.0)	
≥35	8 (72.7)	11 (20.0)	
Median age	37 (28–40)	32 (26–39)	0.002^a^
Gestational age(weeks)	12.4 (11.6–13.3)	12.3 (11.4–13.6)	0.795^a^
Body mass index(kg/m^2^)	21.4 (19.1–24.7)	20.2 (16.6–27.7)	0.465^a^
Nulliparity	4 (36.4)	19 (34.5)	1.000^b^
Gravidity	2.0 (1.0–6.0)	2.0 (1.0–6.0)	0.971^a^
Gender-ratio of fetus(male:female)	5∶6	33∶22	0.507^b^
IVF treatment	5 (45.5)	0 (0.0)	

Values are median (interquartile range) or number (%).

a Mann-Whitney U test,

b Fisher’s exact test.

**Figure 1:**
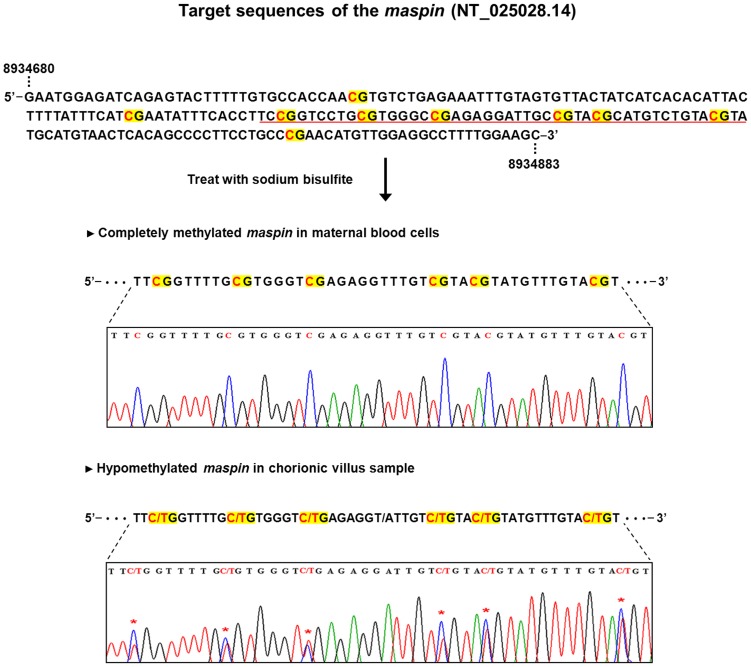
Bisulfite sequencing of the *maspin* promoter in first trimester paired placental tissue and maternal blood cells. The red characters in the sequences indicate methylation CpG sites of the *maspin* gene. Unmethylated CpG sites of *maspin* were detected only in placental tissue. Methylated CpG sites of *maspin* were detected in placental tissue and maternal blood cells.

The tissue-specific epigenetic characteristics of the *maspin* gene revealed by bisulfite sequencing are shown in Figure 1. The *maspin* gene was relatively hypomethylated in the placenta and completely methylated in maternal blood cells.

**Figure 2:**
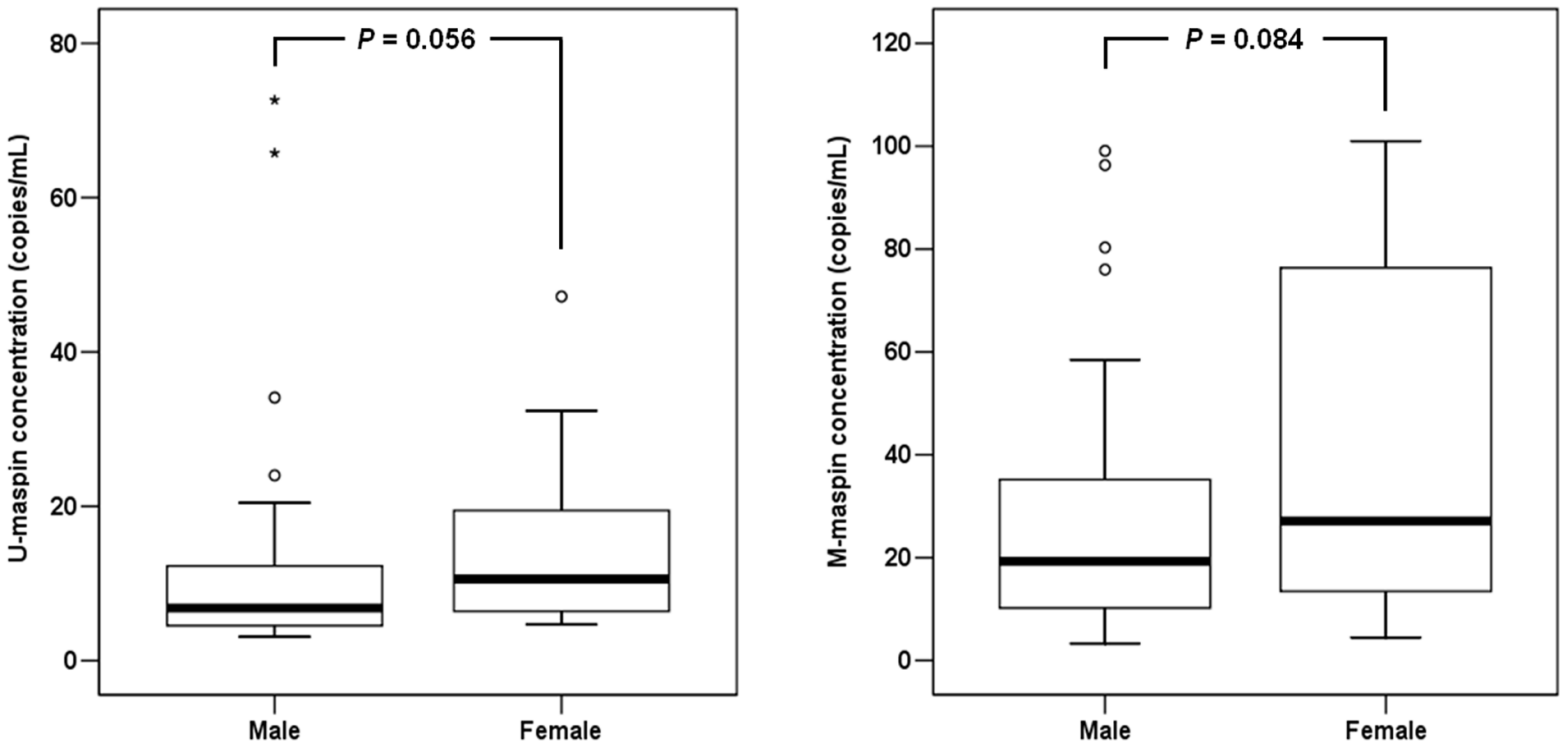
Box plots of U-*maspin* and M-*maspin* concentrations in first trimester maternal plasma according to fetal sex. The upper and lower limits of the boxes represent the 75th and 25th percentiles, respectively. The upper and lower whiskers represent the 90th and 10th percentiles, respectively. The median is indicated by the line in each box. Outliers are indicated by circles and extremes by asterisks.

We analyzed U-*maspin* and M-*maspin* concentrations according to fetal sex. The median concentrations of U-*maspin* were 6.8 (interquartile range: 3.1–72.6) and 10.6 (interquartile range: 4.7–47.2) copies/mL in women bearing male and female fetuses, respectively. The corresponding concentrations of M-*maspin* were 19.4 (interquartile range: 3.3–99.1) and 27.1 (interquartile range: 4.5–101.0) copies/mL, respectively. Concentrations of U-*maspin* and M-*maspin* were not significantly different between fetal sexes (male and female) (*P*>0.05 for both) (Fig. 2).

**Figure 3:**
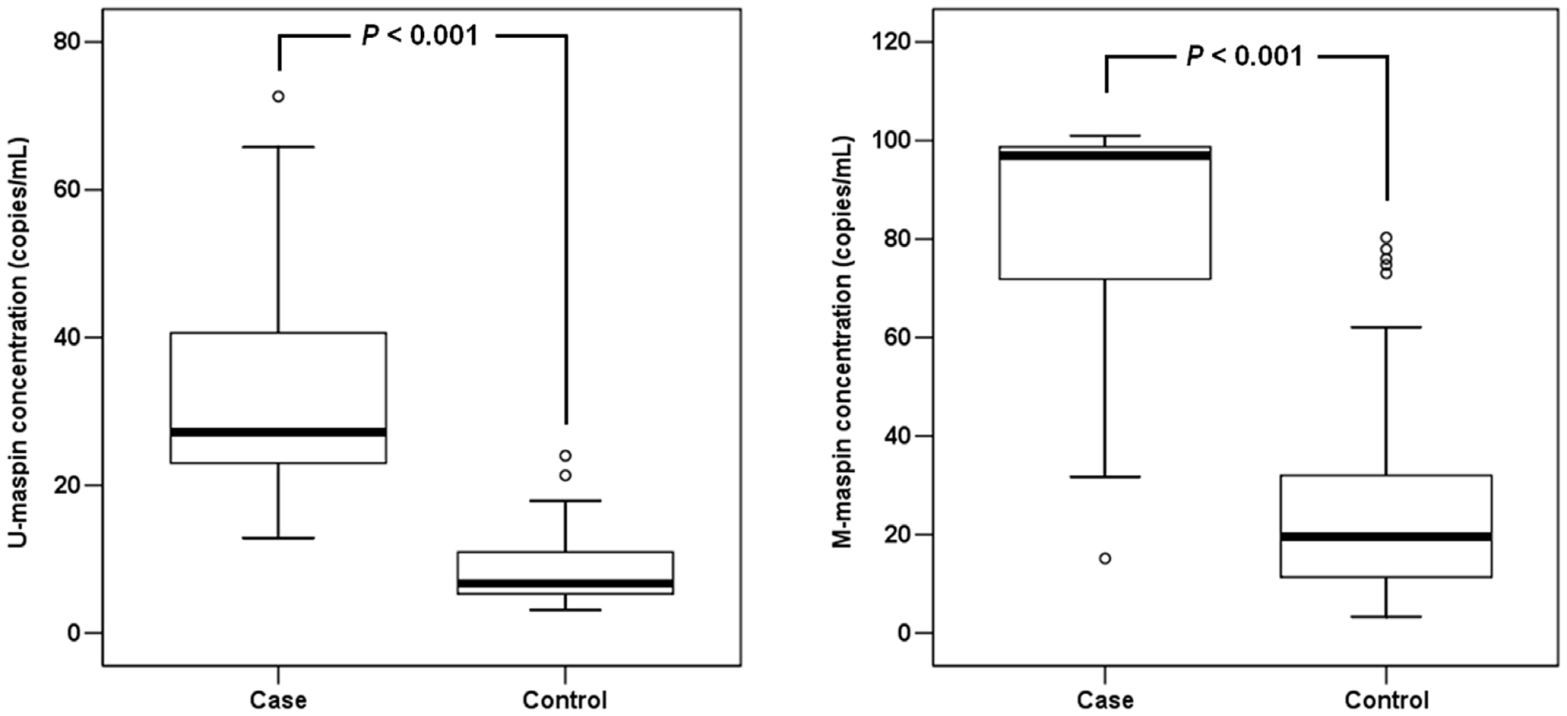
Box plots of U-*maspin* and M-*maspin* concentrations in first trimester maternal plasma from cases and controls. The upper and lower limits of the boxes represent the 75th and 25th percentiles, respectively. The upper and lower whiskers represent the 90th and 10th percentiles, respectively. The median is indicated by the line in each box. Outliers are indicated by circles.

In the analysis according to karyotype, the concentrations of U-*maspin* were significantly different between cases and controls [median 27.2 (interquartile range: 12.9–72.6) vs. median 6.7 (interquartile range: 3.1–24.0) copies/mL, *P<*0.001]. The concentrations of M-*maspin* were also significantly higher in the cases than in controls [median 96.9 (interquartile range: 15.1–101.0) vs. median 19.5 (interquartile range: 3.3–80.3) copies/mL, *P<*0.001] (Fig. 3).

**Figure 4:**
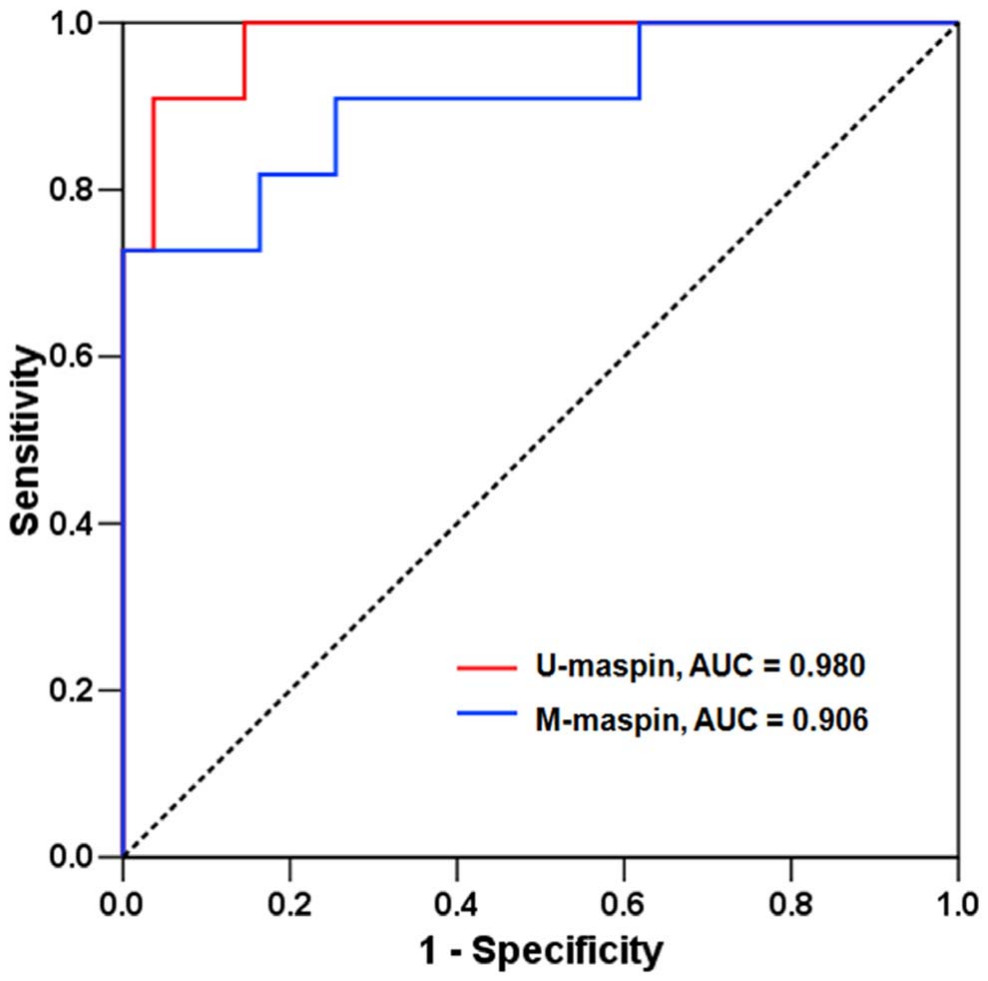
ROC curves of U-*maspin* and M-*maspin* concentrations. The ROC curves of U-*maspin* and M-*maspin* concentrations are represented by red and blue lines, respectively.

To evaluate the accuracy of this approach for non-invasive detection of fetal trisomy 18, ROC curve analyses were performed for each factor (Fig. 4). The AUCs of U-*maspin* and M-*maspin* concentrations were 0.98 (95% CI: 0.95–1.01) with a standard error (SE) of 0.02 and 0.91 (95% CI: 0.79–1.02) with a SE of 0.06, respectively (*P*<0.001 for both). The optimal cutoff value for each factor was set at 90.9% sensitivity, as determined by ROC analysis. The U-*maspin* cutoff concentration of 19.2 copies/mL had a sensitivity of 90.9%, specificity of 96.4%, PPV of 83.3%, and NPV of 98.1% for identifying women carrying a trisomy 18 fetus from women carrying a normal fetus. The M-*maspin* cutoff concentration of 31.5 copies/mL had a sensitivity of 90.9%, specificity of 74.5%, PPV of 41.7%, and NPV of 97.6% (Table 3).

**Table 3:** The utility of measurements for the detection fetal trisomy 18.

	Cutoff	Sensitivity	Specificity	PPV	NPV	ACC
U-*maspin* concentration (copies/mL)	19.19	0.909 (0.636–0.995)	0.964 (0.909–0.981)	0.833 (0.583–0.912)	0.981 (0.926–0.999)	0.980 (0.950–1.011)
M-*maspin* concentration (copies/mL)	31.50	0.909 (0.602–0.995)	0.745 (0.684–0.763)	0.417 (0.276–0.456)	0.976 (0.896–0.999)	0.906 (0.792–1.019)

PPV, positive predictive value; NPV, negative predictive value; ACC, accuracy.

The number in parentheses indicates the 95% confidence interval.

The associations between each factor and the risk of trisomy 18 are shown in Table 4. The risk of trisomy 18 was significantly increased in women with U-*maspin* or M-*maspin* concentration greater than the cutoff values compared with women whose values were lower than the cutoff values [OR 265.0 (95% CI: 21.9–3208.3, *P*<0.001) for U-*maspin* concentration; OR 29.3 (95% CI: 3.4–249.7, *P*<0.001) for M-*maspin* concentration]. After adjusting for potential confounding factors such as maternal age, BMI, and gestational age at blood sampling, the adjusted ORs of U-*maspin* and M-*maspin* concentration were 325.2 (95% CI: 17.9–5903.8, *P*<0.001) and 19.0 (95% CI: 2.1–175.6, *P* = 0.009), respectively. Thus, women with U-*maspin* and M-*maspin* concentration greater than the cutoff values were at higher risk of fetal trisomy 18 in both unadjusted and adjusted analyses, compared with women with concentrations lower than the cutoff values.

**Table 4:** Unadjusted and adjusted OR for fetal trisomy 18.

Factors	Case	Control	Unadjusted OR	*P*	Adjusted OR	*P*
	(n = 11)	(n = 55)	(95% CI)		(95% CI)*	
U-*maspin* concentration (copies/mL)						
Lower than 19.19	1	53	265.0 (21.9–3208.3)	<0.001	325.2 (17.9–5903.8)	<0.001
19.19 or higher	10	2				
M-*maspin* concentration (copies/mL)						
Lower than 31.50	1	41	29.3 (3.4–249.7)	<0.001	19.0 (2.1–175.6)	0.009
31.50 or higher	10	14				

OR, odds ratio; CI, confidence interval.

* Adjusted for maternal age, body mass index, and gestational age at blood sampling.

## Discussion

In this study, we demonstrated the feasibility of applying the U-*maspin* gene as a cell-free fetal DNA marker and the M-*maspin* gene as a cell-free total DNA marker in maternal plasma for the non-invasive prenatal detection of fetal trisomy 18 during the first trimester of pregnancy. We found that U-*maspin* and M-*maspin* concentrations were significantly elevated in the first trimester in pregnant women carrying trisomy 18 fetuses compared with those carrying normal fetuses. Furthermore, pregnant women with high values for U-*maspin* and M-*maspin* were at an increased risk of carrying a trisomy 18 fetus. Therefore, our results suggest that U-*maspin* and M-*maspin* concentrations may be useful biomarkers for non-invasive detection of fetal trisomy 18 using circulating fetal DNA from maternal plasma, irrespective of fetal sex and genetic polymorphisms.

Current methods of non-invasive prenatal screening of trisomy 18 increase the detection rate by integration of nuchal translucency measurement and pregnancy-associated plasma protein-A in the first trimester and various maternal serum biochemical markers, including alpha fetoprotein, unconjugated estriol, human chorionic gonadotrophin and inhibin-A in the second trimester [2]. However, this approach measures epiphenomena associated with the trisomies rather than directly detecting the core abnormality involving chromosomal dosage. Moreover, normal pregnant women with false-positive results undergo unnecessary invasive prenatal diagnostic procedures, such as amniocentesis and chorionic villus sampling, which carry a small but significant risk of miscarriage in normal pregnancies [3]. Therefore, the development of non-invasive prenatal tests for trisomy 18 using cell-free fetal DNA in maternal plasma has been considered as a potential alternative to improve current screening tests and reduce the need for invasive procedures.

They are very few previous reports of the use of epigenetic markers for non-invasive detection of trisomy 18 in maternal plasma. Tong *et al.* reported the novel approach by analyzing the allelic ratio of a polymorphism using a single nucleotide polymorphism (SNP) in the *maspin* gene on chromosome 18 and applied this strategy to NIPT of trisomy 18 [16]. However, the allelic ratio analysis was informative in only 11 among 100 maternal plasma samples tested in the first or third trimester. This approach is therefore limited to fetuses that are polymorphic for the marker and linked SNPs on the target chromosome and needs a combination of multiple SNPs to maximize the population coverage of the test. Recently, Tsui *et al*. described an epigenetic-genetic chromosome dosage approach comparing the ratio of methylated *VAPA-APCDD1* (VAMP (vesicle-associated membrane protein)-associated protein A and Adenomatosis polyposis coli down-regulated 1; chr 18) and *ZFY* (zinc finger protein, Y-linked; chr Y) in euploid and trisomy 18 fetuses [20]. Although this method was discriminative for trisomy 18, its use is limited to the 50% of pregnancies involving male fetuses and therefore needs to be successfully demonstrated with sex-independent markers before it can be used in practice. Moreover, these studies were performed with maternal blood samples that were taken during the late-first to third trimester (15–36 weeks of gestation in Tong *et al*. [8]; 13.3–14.1 weeks of gestation in Tsui *et al*. [20]) and were conducted on small samples (2 cases and 9 controls in Tong *et al*. [16]; 9 cases and 27 controls in Tsui *et al*. [20]). Nonetheless, despite their limitations these previous studies suggest that diagnostic strategies using fetal-specific epigenetic markers have potential for the non-invasive detection of fetal trisomy 18.

In the present study, we evaluated the utility and accuracy of non-invasive detection of fetal trisomy 18 using tissue-specific epigenetic characteristics of the *maspin* gene in maternal plasma during the first trimester of pregnancy. We conducted a nested case-control study controlling for gestational weeks at blood sampling. Furthermore, all samples were obtained during the first trimester (less than 14 weeks of gestation) and the sample number was larger than those of prior studies [16], [20]. Previous findings suggest that the placenta is the major source of cell-free fetal DNA in maternal plasma [11], whereas maternal blood cells are the major source of maternal DNA [12]. On the basis of these results, we used U-*maspin* as an epigenetic signature specific to the placenta to target cell-free fetal DNA, and used M-*maspin* as a cell-free total DNA marker including both cell-free fetal and maternal DNA in first trimester maternal plasma. Although the concentrations of both U-*maspin* and M-*maspin* gene sequences were significantly elevated during the first trimester in pregnancies carrying trisomy 18 fetuses, U-*maspin* was more effective than M-*maspin* for NIPT of fetal trisomy 18. We believe that one possible explanation for these results is the large interindividual variability by the methylation density of maternal cell-free DNA in M-*maspin* gene.

These two factors had relatively high detection rates (90.9% for both) compared with the prior study using the ratio of methylated *VAPA-APCDD1* to *ZFY* (88.9%) [20]. Furthermore, these epigenetic markers have extended the application of cell-free fetal DNA to essentially all pregnancies, regardless of the sex and genetic variations of the fetus. In addition, as a single marker, the sensitivity and specificity of U-*maspin* concentration was higher than, or similar to, those of the combined serum screening test (serum screening markers and/or nuchal translucency measurements) that is generally performed in the first trimester [2], [21].

More recently, massively parallel sequencing (MPS) has been applied to determine the proportional amounts of chromosome 18 DNA molecules in maternal plasma, but showed relatively low diagnostic accuracy compared to trisomy 21 [22], [23]. The main reason that MPS performs poorly for trisomy 18 compared to trisomy 21 is due to GC content bias. Chromosomes with low GC content are underrepresented, whereas chromosomes with high GC content are overrepresented [24]. Chromosome 18 has relatively lower average GC content (39.7%) than chromosome 21 (40.9%) [23]. In the detection of fetal aneuploidy via MPS, the amplification efficiency of the DNA libraries may be non-uniform across sequences with different GC contents, although the form and extent of biases are different among different MPS platforms. Therefore, prior studies reported that the non-uniform representation of each chromosome is more likely to be due to sequencing or alignment bias than biological reasons [24], [25]. In our study, the methylation-based test was not correlated with GC content, but was correlated with CpG content. Moreover, the proportion of U-*maspin* in maternal plasma may be due to biological reasons. As a result, the accuracy for detecting fetal trisomy 18 using U-*maspin* was similar to that of trisomy 21, with a sensitivity of 95% [26]. However, the sensitivity and specificity of U-*maspin* and M-*maspin* concentrations were lower than those for NIPT of trisomy 18 using MPS [22], [23], [27]. Therefore, improvements in the detection rate by applications of other detection methods such as digital PCR or a combination of other epigenetic markers may facilitate the clinical use of these markers.

To the best of our knowledge, this is the first study to estimate the accuracy of non-invasive prenatal detection of trisomy 18 using both cell-free fetal and total DNA markers. Our results demonstrated the feasibility of applying U-*maspin* and M-*maspin* concentrations from circulating fetal DNA in first trimester maternal plasma for the non-invasive prenatal detection of fetal trisomy 18. We suggest that U-*maspin* and M-*maspin* concentrations are useful epigenetic markers for the prediction of fetal chromosomal aneuploidy during the first trimester of pregnancy and might be relevant to many other applications of prenatal testing and monitoring. However, this study confirmed the positive results of NIPT by CVS karyotyping. Thus, we cannot completely exclude the possibility of confined placental mosaicism causing false positive results. Moreover, the use of these epigenetic markers could create ethical and social issues because the results may impact decisions to terminate or continue the pregnancy. Therefore, the use of these markers in clinical situations should be carefully considered and should not be applied in the present clinical setting until more research has been performed. Additionally, this study is limited by its relatively small sample size, therefore further studies in a larger clinical cohort will be needed to validate the clinical utility of measuring *maspin* gene sequences for prenatal assessment of fetal trisomy 18.
